# Transactions of the Atlanta Obstetrical Society

**Published:** 1893-09

**Authors:** 


### TRANSACTIONS OF THE ATLANTA OBSTETRICAL SOCIETY.

ATLANTA, GA., July 15, 1893.

Society called to order by Temporary President, DR. R. R. KIME.

DR. GEO. H. NOBLE read a paper on

### MANAGEMENT OF THE INEVITABLE ABORTIONS.

(SYNOPSIS.)

Is an abortion inevitable? This is determined by the amount and persistence of hemorrhage, escape of liquor amnii, persistence of pain and dilatation.

When we decide an abortion to be inevitable, first cleanse external genitalia and vagina antiseptically. The longer the delay the greater is the risk.

If the dilatation is not complete, apply an antiseptic tampon which will soften the parts and prevent excessive bleeding and enable the operator to prepare for antiseptic evacuation. No woman is safe from septic infection as long as there is a discharge of blood.

Antisepticize vagina and external genitals thoroughly before the application of the tampon, after which patient may be left to prepare for careful evacuation of contents, etc. Upon removal of the tampon the whole or portion of fetal mass may be found on the plug. Douche vagina thoroughly, search uterine cavity with index finger. If it has not been completely cleared, detach fragments with fingers or dull curette. He introduces index finger of right hand into uterine cavity, firmly grasping the fundus uteri through abdominal walls with left hand, and crowds down upon the finger within. If unable to remove contents with fingers, introduces forceps. He uses a pair of forceps shaped like a small pair of obstetrical forceps; (this, however, is rarely necessary, for the finger is the best instrument) or breaks whole mass down and washes it way with a Davidson syringe, then scrapes uterine walls with broad scrape or wire curette. If an anesthetic is necessary he uses ether from force of habit, though chloroform has proven a very safe anesthetic in his obstetrical practice.

Dilatation is of great importance. Up to sixteen weeks of gestation, dilate os sufficiently wide to admit the index finger; after this it must be dilated large enough to introduce two or more fingers. In the first two months the contents may be removed by the curette, but he never feels satisfied without introducing the finger for purpose of exploring the interior of the womb.

In the third month of pregnancy it is often a difficult matter to introduce the finger on account of the length of cervix, there being great increase in the partio-media. He has telescoped cervix on finger and could scarcely pass beyond internal os (in exceptional) cases. At six months the dilatation must be large enough for the admission of two fingers at least, to begin evacuation. Beyond the fourth month dilatation not admitting two fingers does not allow sufficient drainage, and the danger of absorption of septic matter is great, although he has successfully evacuated cases in which dilatation was wide enough to admit only one finger at four and a half months.

In septic cases it is sometimes difficult to determine just the course to be pursued. Some cases will demand decidedly active treatment, while others, perfectly free of fragments or shreds of secundines and suffering from infection of normal discharges, seem only to require the temporizing or douching. Yet under the latter treatment they not infrequently become very grave, and require the most heroic measures, viz., sharp curetting and packing.

The objection to the active treatment in septic cases is that unless thoroughly and completely done the operation is followed by chill and elevation of temperature and the patient made worse for the time being, which is decidedly unpleasant, even though she may improve rapidly afterwards.

If there has not been much absorption, use a dull curette. If the absorption has gone further, the mucous membrane may become thoroughly infiltrated with septic matter, and if not entirely removed will be immediately taken up through the fresh abrasions caused by the dull curette.

He has known cases of absorption to go on with temperature 100° to 101° F., the odor of discharges becoming pronounced, and on examination found os patulous; removed parts of placenta which were left, and douched cavity. In fifteen minutes the patient was seized with rigor and died in a few hours. He saw another case in which the discharge was fetid and the cavity improperly curetted. The patient died almost as soon as in the preceding case.

The lacerations must not be overlooked, as damage to cervix in the way of tears, etc., opens up new avenues for infection, and unless all particles are removed from the uterine cavity the danger is increased.

In curetting great care should be used to clean the angles of the womb, using for the purpose a narrow scoop. The other instrument must have a wide surface so as to avoid the liability of piercing the uterine walls, and leaving strips of mucosa untouched. The best instrument is the finger, with a banjo thimble, which is like an elongated finger nail. With this scrape carefully uterine walls, and see that there is no obstruction to drainage. In simple aseptic cases the dilatation is sufficient, but after thorough removal of the mucous membrane gauze drainage should be employed.

SEPTIC CASES.—The uterus in its normal condition is a closed cavity, and the fluids escape naturally or by expulsion. After infection, the uterus becomes soft and bag-like. The uterine contractions being diminished or abolished, there is a tendency to an accumulation of the secretions, the removal of which must produce a vacuum unless some foreign substance takes its place, hence the necessity of washing or draining with gauze when the womb is relaxed. When we curette it is better to do a little unnecessary work than not enough. Bear in mind the fact that in using the sharp curette there is danger of scraping a hole in the uterus.

The expectant plan of treatment of septic cases looks like malpractice, but when the membrane is scraped off there is great risk of further absorption through fresh abrasions unless the work is thorough, hence the adherence to the old plan of treatment by many practitioners.

*Case.*—Wife of a physician. Small piece of placenta left in cavity, second day patient had chill, temperature 107° F., pulse 160. He removed piece of placenta with dull curette, no anesthetic, washed out cavity with bichloride solution $\frac{1}{2000}$, which was followed by chill and fever. Next day the husband douched the cavity and accidently pressed syringe nozzle firmly against fundus, which was followed by chill and fever. He again curetted, but removed no fragments or shreds, which was also followed by chill and fever. Dr. N. advised no further curetting and adopted expectant plan of treatment, douching cavity through soft instrument. Patient recovered without high elevation of temperature, chills, etc.

The different tissues seem to take up the poison with different degrees of rapidity. Cellular tissue takes it up more rapidly than the placental site. The vessels are dilated, lymphatics enlarged and absorption takes place rapidly.

*Case.*—An old physician, with sore on finger, delivered fetus Sunday morning, leaving placenta in uterus. Came back Monday and renewed efforts to remove it but failed. Dr. N. was summoned Monday at noon and found temperature 104° F., pulse 160, cervix slightly lacerated, vagina torn from cervix to perineum and uterus very irritable. She had been given ergot. Monday, 12 M., placenta was removed, being very offensive, and uterus thoroughly cleansed. The cavity was then douched with gallons of hot bichloride water and lacerations closed, but patient was so thoroughly intoxicated with septic matter that she died the next day.

Complications may be almost anything that occurs at full term delivery and must be treated as such.

Dilatation.—Five or six years ago a distinguished obstetrician was asked that if he had a case of septic intoxication without dilatation would he dilate? He avoided an answer, but to-day could doubtless answer in the affirmative.

If uterus is soft, dilate with fingers, first introducing one finger, then another, drawing down the internal os with it as the next finger is introduced, until amount of dilatation desired is secured. In the earlier months Goodell's dilator is the most common instrument in use, but this bruises the tissues too unequally. Cylindrical plugs are better. Gauze packing is good, if plenty of time is to be had.

However, packing is not always a safe measure, as bleeding may go on and result in concealed hemorrhage and rupture of uterus if very near the end of gestation.

Induced abortions are to be treated in the same way—that is, by forcible dilatation. His results have been good where this became necessary. He does not believe in catheters or tents for this purpose. The operation is put off as a last resort, and why wait for the woman to exhaust her strength and render the absorption of septic matter more likely? The pregnant uterus is very easily dilated. In his last case he dilated, cleaned out cavity and put woman in bed in fourteen minutes.

DISCUSSION.

DR. KIME: I have been interested very much in this subject and have some positive opinions as to the treatment of inevitable abortion which will soon appear in a paper now in the hands of the printer. (See page 85.) When abortion is inevitable and fetal structures are yet in the uterus, the aseptic vaginal tampon is indicated, letting it remain twelve to eighteen hours. On removal, if abortion is not completed, may tampon a second time for a shorter period, under proper antiseptic precautions.

*After* fetal structures have passed off with maternal retained, the use of the tampon is a question of doubt with some, for it serves to prevent drainage, dams up blood in the uterus with tendency to force it out Fallopian tubes, thus favoring complications.

Adopt rapid dilatation with steel dilators under antiseptic precautions, and then by use of curette forceps and curette the uterus can be readily and efficiently emptied. I confess I have not been as successful in the use of the *finger* for this purpose as some; I may not be as dextrous. Hence my preference for curette forceps and curette. After clearing the uterine cavity, tampon with gauze or throw into uterus (with cervix dilated) ʒ ss to ʒ i camphorated phenique; that is, equal parts of gum camphor and carbolic acid rubbed together. It is the best agent I know of to check putrefaction and pus formation.

The results of such treatment will depend on whether we have putrid infection or septic infection to deal with. In putrid infection, the process is slower, developing gradually, indicated by slight increase of pulse each day, followed by gradual increase of temperature, furred tongue and headache. In such cases clearing and disinfecting the uterine cavity gives most marked and beneficial results.

In *septic* infection the onset is more rapid; patient usually has a chill followed by high pulse, elevated temperature, and is due to introduction of septic germs, which multiply rapidly, enter circulation and lymphatics, setting up new foci of development and complications. By use of curette and antiseptics those beyond uterine cavity and endometrium cannot be reached, and other lines of treatment must be adopted after first clearing and disinfecting uterine cavity. I never use tents, nor does Goodell steel dilators dilate internal os sufficiently. It has been said an infected uterus had a relaxed os, but I have not found it so in my practice. I do not wait for offensive odor of discharges, for some of the worst cases I ever saw had no odor.

Dr. Hardon: The subject has been well covered in the paper presented this evening. I think the whole matter is dependent upon certain general principles. These may be stated as follows: To completely empty the uterus, and to prevent septic infection.

If abortion is inevitable, any treatment must have as its object the emptying of the uterus, and if this is necessary, the sooner it is accomplished the better. I fail to see the use of temporizing if the abortion be inevitable, thereby lengthening the time that the patient is exposed to the two great sources of danger, viz., hemorrhage and septicemia.

The first duty of the attending physician is to empty uterus as oon as he is called. The os uteri in the majority of cases is not sufficiently dilated for the thorough evacuation of the contents of the uterus, and must first be accomplished. In some cases this can be done by the fingers, but in most cases in my experience, the fingers cannot accomplish the dilatation desired, and I resort to use of steel dilators. This method requires from fifteen to twenty minutes for its accomplishment without bruising the parts too much. Having accomplished dilatation, I then introduce the index finger. I find the finger is not the best instrument for the removal of the ovum, for I cannot get sufficient grasp of the structures to be removed with my finger. I do, however, get an idea of the location of the parts to be removed by the introduction of the finger, and can more intelligently direct the instrument which I use. Having located the material to be removed, I then introduce Emmett's fenestrated curette forceps and remove.

After removal, unless I chance to remove the ovum entire, it is my custom to curette carefully the uterus, being careful, as Dr. Noble previously said, to cleanse thoroughly horns of uterus. I then irrigate with a 1–5000 bichloride of mercury solution, believing this to be strong enough for all purposes and not so likely to produce the toxic effects as the stronger solutions. I sometimes drain with gauze, where I am certain that all material has been removed, for it must be remembered that gauze only drains fluids—no blood or solid matter. This may cause a retention of clots, etc., and do away with the object desired. In cases of septic or putrid infection with quick pulse, high temperature and other usual signs of such trouble, the course is the same, viz.: Empty uterus thoroughly and wash out. This mode of treatment best fulfills the old injunction, *tuto, cito, jucunde.*

This puts the patient beyond the reach of septicemia and fulfills all requirements, obviating the two great dangers, hemorrhage and septicœmia.

---

DR. ALBERT BRUBAKER says that *spasmodic croup* in children, coming on suddenly at night, is often due to impaired digestion brought on by eating some heavy food just before retiring. If the stomach in these cases be emptied by an emetic, it will be found that the croup will also disappear.—*Cal. and Clin. Record*

